# RNA-Sequencing Analysis of Adrenocortical Carcinoma, Pheochromocytoma and Paraganglioma from a Pan-Cancer Perspective

**DOI:** 10.3390/cancers10120518

**Published:** 2018-12-15

**Authors:** Joakim Crona, Samuel Backman, Staffan Welin, David Taïeb, Per Hellman, Peter Stålberg, Britt Skogseid, Karel Pacak

**Affiliations:** 1 Department of Medical Sciences, Uppsala University, Akademiska Sjukhuset ing 78, 75185 Uppsala, Sweden; staffan.welin@medsci.uu.se (S.W.); britt.skogseid@medsci.uu.se (B.S.); 2 Section on Medical Neuroendocrinology, Eunice Kennedy Shriver National Institute of Child Health and Human Development, National Institutes of Health, 10 Center Drive, Building 10, Room 1E-3140, Bethesda, MD 20892, USA; karel@mail.nih.gov; 3 Department of Surgical Sciences, Uppsala University, Akademiska Sjukhuset ing 70, 75185 Uppsala, Sweden; samuel.backman@surgsci.uu.se (S.B.); per.hellman@surgsci.uu.se (P.H.); peter.stalberg@surgsci.uu.se (P.S.); 4 Department of Nuclear Medicine, La Timone University Hospital, European Center for Research in Medical Imaging, Aix Marseille Université, 13385 Marseille, France; David.TAIEB@ap-hm.fr

**Keywords:** pheochromocytoma, paraganglioma, adrenocortical carcinoma, adrenal tumor, pan-cancer analysis, neural crest, neuroendocrine

## Abstract

Adrenocortical carcinoma (ACC) and pheochromocytoma and paraganglioma (PPGL) are defined by clinicopathological criteria and can be further sub-divided based on different molecular features. Whether differences between these molecular subgroups are significant enough to re-challenge their current clinicopathological classification is currently unknown. It is also not fully understood to which other cancers ACC and PPGL show similarity to. To address these questions, we included recent RNA-Seq data from the Cancer Genome Atlas (TCGA) and Therapeutically Applicable Research to Generate Effective Treatments (TARGET) datasets. Two bioinformatics pipelines were used for unsupervised clustering and principal components analysis. Results were validated using consensus clustering model and interpreted according to previous pan-cancer experiments. Two datasets consisting of 3319 tumors from 35 disease categories were studied. Consistent with the current classification, ACCs clustered as a homogenous group in a pan-cancer context. It also clustered close to neural crest derived tumors, including gliomas, neuroblastomas, pancreatic neuroendocrine tumors, and PPGLs. Contrary, some PPGLs mixed with pancreatic neuroendocrine tumors or neuroblastomas. Thus, our unbiased gene-expression analysis of PPGL did not overlap with their current clinicopathological classification. These results emphasize some importances of the shared embryological origin of these tumors, all either related or close to neural crest tumors, and opens for investigation of a complementary categorization based on gene-expression features.

## 1. Introduction

The adrenal gland is derived from two components that are developmentally and physiologically distinct: Cells of the adrenal cortex are derived from mesoderm and are characterized by steroid metabolism. Neuroectodermally derived adrenal medulla is encircled by the adrenal cortex and contains neuroendocrine (chromaffin) cells synthesizing catecholamines [1]. These characteristics are retained in adrenal neoplasms that are classified accordingly by the World Health Organization into tumors of the adrenal cortex and tumors of chromaffin cells of the adrenal medulla and extra-adrenal paraganglia (PPGL) [2]. Molecular techniques further stratifies these tumors into distinct categories [3,4]. The adrenal cortex derived adrenocortical carcinoma (ACC) is separated into three subgroups; cluster of clusters 1–3 with differences in steroid differentiation, cell proliferation, DNA methylation and spectrum of genetic driver events [5,6]. Similarly, PPGLs are separated into 4 groups named after their molecular characteristics: pseudohypoxia related to succinate dehydrogenase or *VHL/EPAS1* disturbances, wnt-altered and kinase-signaling pathways [7,8,9].

New approaches and methods for analysis of molecular pan-cancer datasets may obtain novel insights into the characteristics of a wide range of neoplasms in a single experiment. Their results can be used to test whether the current clinicopathological classification of a particular tumor remains relevant on a molecular level [10,11]. Current state of the art and views suggest that a majority of tumor types categorize accordingly to their established clinicopathological classifications in such pan-cancer analyses [11]. However, an alternative scenario where new molecular analyses proposed a new disease categorization has been shown for some cancers [12]. One example is esophageal carcinoma where the squamous cell subtype resembled squamous cell carcinomas of other organs, whereas the esophageal adenocarcinoma clustered with gastric adenocarcinoma [12]. Thus, we hypothesized that the differences between subgroups of ACC and PPGL could be significant enough to support an updated classification of these tumors. One example could be the pronounced pseudohypoxia phenotype that is shared among some PPGLs and other neural crest tumors. We used a pan-cancer analysis, that allowed for an unbiased clustering of tumors based on gene expression data, to test this hypothesis.

## 2. Results

### 2.1. Aim 1: To Determine if ACC and PPGL Show Integrity in a Transcriptomic Pan-Cancer Context

**Figure 1:**
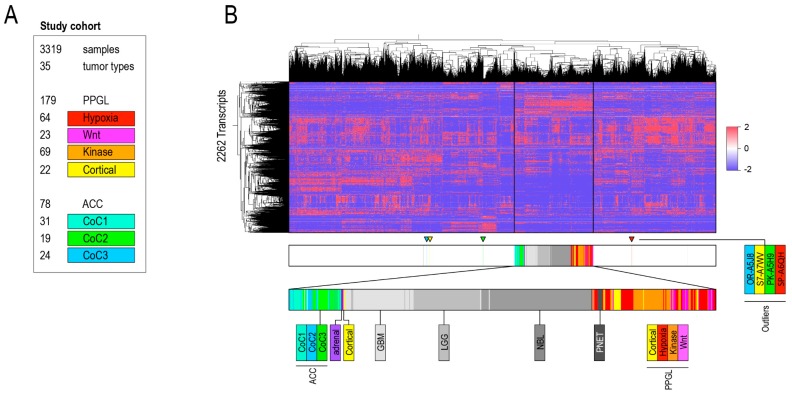
Pan-cancer dataset and transcriptomic classification. (**A**) Pan-cancer analysis dataset and pipeline. Results from bioinformatics pipeline 1. (**B**) Unsupervised hierarchal clustering of RNA-seq data from 3319 TCGA and TARGET samples annotated for cancer type processed by bioinformatics pipeline 1. Abbreviations; ACC, Adrenocortical Carcinoma; GBM, Glioblastoma Multiforme; LGG, Brain Lower Grade Glioma Neuroblastoma; PNET, Pancreatic Neuroendocrine Tumor; PPGL, Pheochromocytoma and Paraganglioma; Cortical, Cortical Admixture PPGL; Hypoxia, Pseudohypoxic PPGL; Kinase; Kinase signaling PPGL and Wnt, wnt-altered PPGL.

To address whether the current clinicopathological classification of ACC and PPGL remains relevant in a transcriptomic pan-cancer context, we performed unsupervised clustering and principal component analyses. RNA-seq data from 3319 tumor samples of 35 different categories from the Cancer Genome Atlas (TCGA) and Therapeutically Applicable Research to Generate Effective Treatments (TARGET) (Figure 1A, Table 1) were included.

Cases were grouped by TCGA tumor category and genes with high variability between tumor categories were extracted. Dendrograms of unsupervised clustering showed integrity of both ACC and PPGL which formed two separate clusters (Supplementary Figures S1A–C and S2). In the second series of experiments we analyzed the dataset on a per sample basis. Genes with a variable expression in-between 3319 cases were selected and analyzed with unsupervised clustering. The pan-cancer dendrogram recapitulated previous findings described by Hoadley et al. including clustering accordingly to organ (e.g., kidney, gastrointestinal tract) and cell of origin (e.g., squamous cell cluster) (Supplementary Figure S3A–C) [10,11]. Except for a few outliers, ACC remained a homogenous group whereas PPGL mixed with pancreatic neuroendocrine tumors (PNETs) in 2 out of 3 unsupervised clustering experiments (Figure 1B, Supplementary Figure S3A–C). In order to investigate the robustness of these results, we designed a second bioinformatics pipeline that used different software for sample selection, identification of genes with variable expression and unsupervised clustering. These results validated that ACC formed one homogenous cluster whereas a group of kinase signaling PPGL mixed with a group of neuroblastoma (NBL) (Supplementary Figure S4A,B). Inspection of the clustering dendrogram revealed sub-separation of ACC Cluster of Clusters 1 (COC1) from COC2 and 3. In PPGL, kinase signaling tumors separated either gradually (bioinformatics pipeline 1) and distinctively (bioinformatics pipeline 2) from pseudohypoxic and wnt-altered tumors (Figure 1, Supplementary Figure S4A).

#### Detailed Analysis of ACC and PPGL Outliers

**Figure 2:**
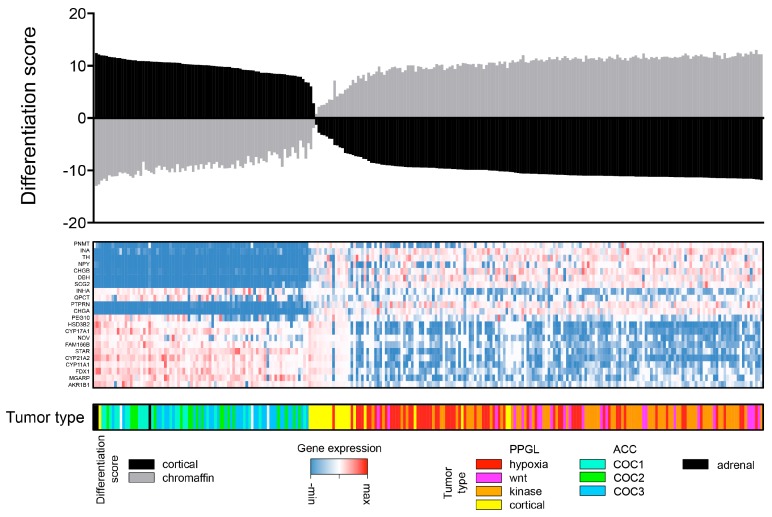
Chromaffin and cortical cell differentiation. Cortical and chromaffin cell differentiation of adrenocortical carcinoma (ACC), pheochromocytoma and paraganglioma (PPGL) and adrenal gland samples. Each column represents a unique sample that was ordered according to cortical cell differentiation. From above: differentiation scores for adrenal cortex (black) and adrenal medulla (grey). Middle; heatmap with expression values of genes representing chromaffin cells (upper half) and cortical cells (bottom half). Bottom: annotation of sample type accordingly to PPGL and ACC molecular subtypes. COC, Cluster of Clusters; Cortical, Cortical Admixture PPGL; Hypoxia, Pseudohypoxic PPGL; Kinase; Kinase signaling PPGL and Wnt, wnt-altered PPGL.

ACC and PPGL samples that clustered outside their disease group in both bioinformatics pipelines were carefully examined. There was one ACC, OR-A5J8, of sarcomatoid type with 100% purity that clustered among sarcomas (SARC). It showed a cortical differentiation score of 7.9, 4th lowest among ACCs (Figure 2). Analysis of all ACCs and all SARCs available in TCGA that showed that OR-A5J8 clustered to the SARC group (Supplementary Figure S5A–C). The second sarcomatoid ACC available in the TCGA dataset clustered among ACCs.

One PPGL mixed into the ACC cluster: TT-A6YO of the cortical admixture subgroup with 66% purity. It had a cortical differentiation score of 12 (higher than all ACCs) and a chromaffin differentiation score of −12 (lower than all PPGLs). In TCGA, this sample was noted to have cortical cells through histopathological analysis [9]. Two additional PPGLs clustered outside the main group, both were pheochromocytomas of the cortical admixture subgroup (one had admixture of adrenocortical cells by histopathology) and their tumor purity was 54 and 100%, respectively. Cortical differentiation score was 6.1 and −3.5, respectively (2nd and 8th highest among PPGL). Chromaffin differentiation score was −5.9 and 2.9, respectively (2nd and 8th lowest among PPGL). Thus, we have concluded that sample misclassification and infiltration of non-tumor cells are two likely explanations for samples that consistently clustered outside ACC or PPGL main groups (Figure 2).

PNETs (bioinformatics pipeline 1, Section 4.1) and NBL (bioinformatics pipeline 2, Section 4.2) infiltrated the PPGL group (Figure 2, Supplementary Figures S3A–C and S4A,B). Inspection of samples that clustered outside PPGL main group in one of two bioinformatics pipelines revealed a pattern with enrichment of either dopamine secreting thoracic PPGL with metastatic disease (bioinformatics pipeline 1) or kinase signaling PPGL (bioinformatics pipeline 2).

### 2.2. Aim 2: To Identify with Which Cancers ACC and PPGL Show Similarities

**Figure 3:**
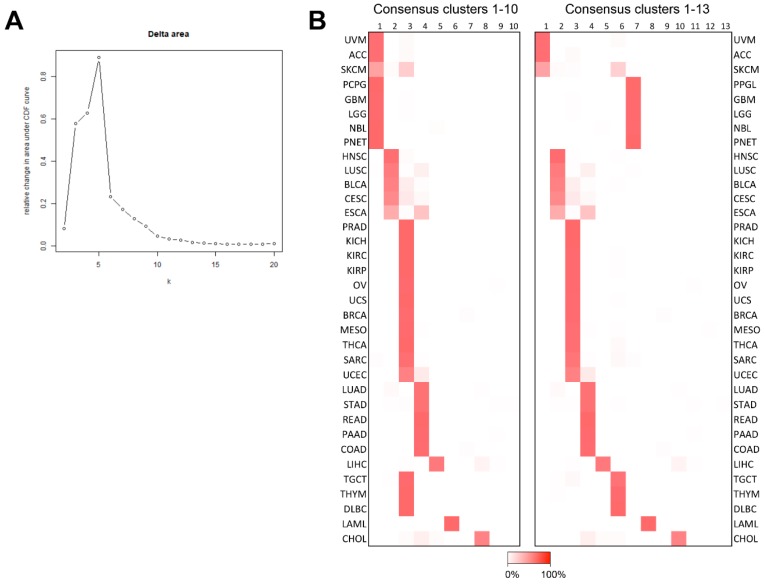
Pan-cancer consensus clustering. Unsupervised consensus clustering of 3319 TCGA and TARGET samples annotated for their specific cancer type. (**A**), Delta CDF plot with information on the additional explanatory power provided through increasing the number of clusters. Y-axis, relative change in the area under CDF curve and y-axis; *k*, the number of consensus clusters. (**B**), Proportion of cases assigned to the different clusters ranging from 0% (white) to 100% (red) in both 10 and 13 clusters. X-axis, consensus cluster numbers and y-axis, a cancer type.

Adrenocortical carcinoma, glioblastoma multiforme (GBM), low grade glioma (LGG), NBL, PNET, and PPGL clustered together in the 6 of the 8 experiments performed in the bioinformatics pipelines (Supplementary Figures S1A–C, S2, S3A–C and S4A,B). The relative associations within this group of tumors varied between the different experiments. To exclude that the inclusion of ACC and PPGL molecular subtypes skewed the results of the per-TCGA tumor category analysis, unsupervised clustering was repeated without separation of ACC and PPGL into molecular subgroups. This experiment showed similar results (Supplementary Figures S6A–C and S7). We also investigated whether a signal of adrenocortical cells in PPGL could influence the outcome and thus, we removed all pheochromocytomas. Unsupervised clustering showed that ACC remained among neural crest tumors (Supplementary Figure S8). Consensus clustering experiments validated an ACC, GBM, LGG, NBL, PNET, and PPGL cluster that also included skin cutaneous melanoma (SKCM) and uveal melanoma (UVM) (Figure 3A,B, Supplementary Figure S9A–C). As the number of permitted clusters was increased, this cluster was partitioned into: (1) GBM, LGG, NBL, PNET, and PPGL as well as (2) ACC, SKCM and UVM (Figure 3B). These results overlapped previous pan-cancer findings where PPGL grouped together with either GBM and LGG or NBL [11]. 

The clustering of ACCs to neural crest tumors was an unexpected finding lacking an obvious explanation [11,43]. In order to identify the gene expression profile that drove these results, we have identified transcripts that were able to discriminate ACC, GBM, LGG, NBL, PNET, and PPGL from the remaining tumors. A total of 78 transcripts showed an AUC of >0.9. Fifteen of these fulfilled the following criteria: 2-fold higher expression in ACC compared to remaining tumors (pan-cancer minus GBM, LGG, NBL, PNET and PPGL) and no less than 0.1-fold difference in expression compared to GBM, LGG, NBL, PNET, and PPGL. Of these 15 genes, 14 had lower expression in ACC compared to neural crest tumors (Supplementary Table S1a). There were no shared molecular hallmarks between ACC to the group of GBM, LGG, PNET, and PPGL detectable through annotation with gene-ontology information (Supplementary Table S1b).

#### A Separate Pan-Glioma-Neuroendocrine Tumor Cluster Analysis

**Figure 4:**
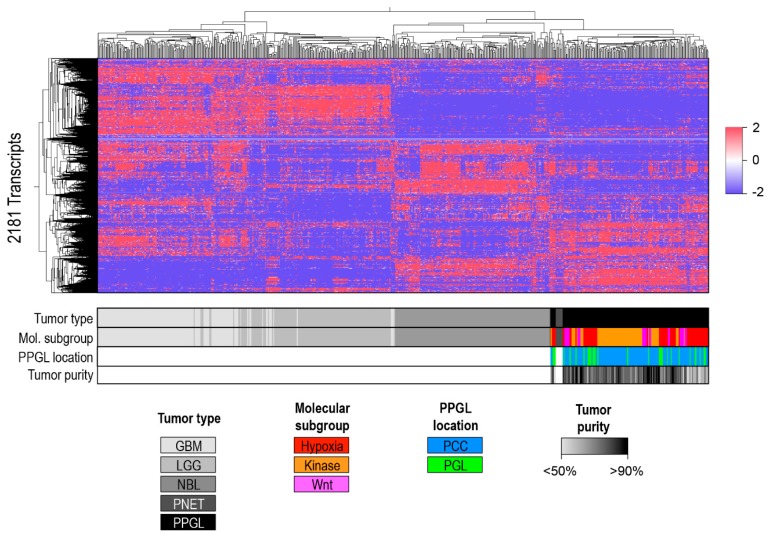
Pan-glioma-neuroendocrine tumors cluster. Unsupervised clustering of RNA-seq data of GBM (*n* = 150), LGG (*n* = 150), NBL (*n* = 156), PNET (*n* = 8) and PPGL (cortical admixture excluded, *n* = 152) processed by bioinformatics pipeline 1.

In the previous analyses we found that GBM, LGG, NBL, PNET, and PPGL form a group of tumors with overlapping transcriptomic profiles. We further analyzed this neural crest group using unsupervised clustering and principal component analysis after removal cortical admixture PPGLs (total *n* = 152) to reduce signal from non-chromaffin cells. To balance the size of the different groups, GBM and LGG were restricted to 150 samples each. Results showed a separation into two clusters, one consisting of low and high grade gliomas and a second including NBL, PNET, and PPGL (Figure 4, Supplementary Figures S10A–C and S11A–C).

## 3. Discussion

In this study we used a pan-cancer model to investigate the degree of overlap between unbiased gene-expression clustering to the current clinicopathological classifications of ACC and PPGL. A second aim was to investigate which other cancer types these two diseases show similarity to. We found that ACC was a homogenous transcriptomic group that showed a surprising association with neural crest derived tumors. PPGLs mixed with either pancreatic NETs or NBL. In addition, it also clustered together with GBM and LGG as well as ACC.

The unique aspect of this study is the combination of two datasets that together has a high number of samples from many different tumor types. The included data provides a very comprehensive characterization of gene expression that has the highest standard for quality control. Specifically, we used the publically available TCGA and TARGET resources to include 3319 tumor samples originating from 35 different tumor types. The most important and novel aspect came from merging a large number of NBLs (TARGET cohort) with other neural crest tumors, including GBM, LGG, PNET, and PPGL (TCGA cohort).

Our first aim was to determine whether findings from an unsupervised clustering of a pan-cancer gene expression dataset overlapped current clinocopathological classifications of ACC and PPGL. Through eight different unsupervised clustering experiments as well as principal component analysis and consensus clustering, we found that both ACCs and PPGLs were relatively homogenous groups of diseases, but that PPGLs mixed with either NBLs or PNETs. As such, our unbiased clustering based on gene expression features did not fully overlap with the current clinicopathological classification of PPGL. This was similar to previous findings related to pancreatic and small intestinal NETs, where clustering by gene expression data revealed mixing of a minority of tumors independently of their primary location [44]. Future studies aggregating NETs from many different primary sites could be used to test whether this group of diseases could use gene-expression data to form a complementary classification.

Our second aim was to identify with which tumor types either ACC or PPGL transcriptomes show similarities to. Our findings corroborate previous pan-cancer studies that identified similarities between PPGLs to both NBLs as well as to low and high grade gliomas (GBMs and LGGs) on the transcriptomic level [11,43]. However the associations of ACC to these cancers differed from a recent pan-cancer study where ACC clustered together with chromophobe renal cancer [11]. We failed to identify a distinct gene expression signature that drove clustering of ACCs to the neural crest group in our experiments. Our conclusion from these observations is that ACC, in relative terms, is more similar to neural crest tumors than other entities included in this pan-cancer study. However, in absolute terms, the similarities between ACC and neural crest tumors are likely not strong. We must note that although the cortical cell has its unique features related to steroid synthesis and metabolism, certain aspects of the ACC genetic landscape overlaps with that of neural crest tumors. This includes presence of both telomerase activation and alternative lengthening of telomeres due to ATRX or DAXX truncation as well as somatic or germline driver mutations in *MEN1* [5,6,45,46]. Another interesting conclusion from this study is that ACC did not cluster with tumors originating from gonadal cells (ovarian serous cystadenocarcinoma and testicular germ cell tumors) with which it shares its developmental origin. This improved knowledge of disease relationships may ultimately be used to motivate cautious extrapolation of results from more extensively studied diseases such as GBM, LGG, and NBL to a rare tumor type such as PPGL that lacks both representative disease models and curative systemic treatment options.

Our analysis validated previous findings in pan-cancer studies including separation tumors accordingly to organ (kidney, gastrointestinal adenocarcinomas etc.) and cell of origin (squamous cell cancers, including separation of esophageal carcinoma). But when interpreting our results some weaknesses should be acknowledged: only one group of neoplasms derived from adrenal cortex (ACC) was included compared to four different diagnoses and 5-fold higher number of samples of GBM, LGG, NBL, PNET and PPGL. Another weakness is that the transcriptomic data was generated from tissue homogenates that do not allow for separation of tumor and non-tumoral cells. Third, our method for transcript selection is likely to select gene expression patterns that are specific to cell-of-origin. As show by Creighton et al., cell-of-origin specific transcripts can be filtered out using an alternative bioinformatics strategy, resulting in a separation according to disease driving mechanisms and a very different clustering of tumors [47]. Finally, our study used only one class of molecular data. As already demonstrated in a pan-cancer analysis of >10000 tumors included in the TCGA consortium, a pan-molecular analysis have the potential to provide additional insights [11].

## 4. Methods

TCGA and TARGET datasets are well suited for comparative studies due to (1) the high number of samples across different disease categories, and (2) the extraordinary standardization in data generation including tissue collection, genome analysis as well as bioinformatics processing. All cases were annotated accordingly to established clinicopathological classifications used in the TCGA and TARGET cohorts (Table 1). ACCs and PPGLs were further annotated accordingly to the molecular classifications proposed in the respective TCGA projects [5,9]. We selected mRNA expression as a proxy of tissue biology due to the gene-specific correlation between mRNA and protein levels [48]. This study falls under an approval from the regional Ethics Committee in Uppsala (544/2015).

### 4.1. Bioinformatics Pipeline 1

#### 4.1.1. Sample Selection and Annotation

Publicly available level 3 data; RNA-seq V2 and clinical annotations available through TCGA and TARGET consortiums were downloaded 2017-08-23 from Genomics Data Commons https://gdc.cancer.gov (Table 1). In the pan-cancer experiments each cohort was restricted to a maximum of 100 samples in order to limit the size for the dataset and to balance the relative weight in-between subgroups. All samples were included for two cohorts; PPGLs (*n* = 179) and NBL (*n* = 156). A total of 8 samples with histopathology of neuroendocrine tumors were included from the pancreatic ductal adenocarcinoma project and annotated as PNET [31]. Samples from primary tumors (01A) were prioritized. For RNA-seq analysis FPKM normalized files were selected and annotated on a per transcript basis for further analysis. Cases were annotated by (1) TCGA tumor type, and (2) sample type (primary tumor, metastasis or normal tissue). Molecular clusters defined by primary TCGA publications were used; ACC, cluster of clusters 1–3; and PPGL, pseudohypoxia, wnt-altered, kinase signaling, and cortical admixture [5,9]. ACC samples without any molecular subtype had a COC value randomly assigned; PPGL samples without a molecular subtype were assigned identical value of tumor samples available from the same patient. Tumor purity values were extracted from the primary publications for PPGL and ACC [5,9]. Three samples from normal adrenal was available through the PPGL cohort and annotated as “Adrenal” [9].

#### 4.1.2. Unsupervised Clustering

Files were imported into the Subio Platform version v1.21.5074 (Subio Inc, Kagoshima, Japan, https://www.subioplatform.com). FPKM transcript counts were subjected to Log2 normalization and genes with low expression values (mean FPKM < 1) were discarded. Genes with high standard deviation of expression in-between (1) TCGA categories, or (2) individual samples were selected for further analysis, three different datasets with different standard deviation thresholds was used for each unsupervised clustering experiment. Unsupervised clustering was performed using a Spearman test as the distance metric. Subio Platform raw data figures were imported into Microsoft PowerPoint (Microsoft Inc, Redmond, WA, USA) and edited for improved readability.

### 4.2. Bioinformatics Pipeline 2

#### 4.2.1. Sample Selection and Annotation

FPKM and raw count files release 10.0 were downloaded between 2018-02-05 and 2018-02-08 from https://gdc.cancer.gov. Cohort size and annotation was performed identical to previous experiments: all NBL, PNET and PPGL samples were included. ACC that were not assigned to a COC in the original publication were assigned as N/A. For other cohorts, a maximum of 100 samples were selected through a random process that was independent to previous experiments. 

#### 4.2.2. Unsupervised Clustering

Sample based clustering: All genes with an index of dispersion (variance/mean) with at least 60 were included. This cutoff was arbitrarily selected to obtain an appropriate number of genes. Values were log2-transformed, using an offset of 1 in order to avoid errors for any samples with FPKM-values of 0 for any of the included genes. A heatmap was generated and clustering based on the Euclidean distance was performed using the heatmap.2 function in the *gplots* R package [49,50,51].

TCGA category-based clustering: The mean expression of each of the included genes in each tumor type was calculated on the basis of the selected samples and heatmap generation and clustering was performed as described.

### 4.3. Consensus Clustering

The dataset comprising 2262 genes identified through extraction of mRNAs with a high variance in expression in-between samples through bioinformatics pipeline 1 was used. Consensus clustering was performed using the *ConsensusClusterPlus* R package [52]. One thousand iterations were performed with a sample inclusion probability of 0.8 and an item inclusion probability of 1. The number of clusters was selected based on inspection of the Delta CDF plot.

### 4.4. Interpretation of Results

Supported by the findings of Hoadley et al. [10] we considered two principle outcomes of the experiments: (1) Concordance between the clinicopathological/anatomic and molecular classification if ACC and PPGL cluster into two homogenous groups, or (2) discordance if ACC or PPGL cluster with other tumor types into intermixed groups or if ACC or PPGL are separated across multiple different clusters.

### 4.5. Multidimensional Scaling Plots

The raw count files of the included samples were processed with the *voom* function in the *limma* R package. Multidimensional scaling-plots were generated using the *plotMDS* function and the *ggplot2* R package.

### 4.6. Adrenal Medulla and Cortex Differentiation Scores

A dataset of 2262 genes identified through extraction of mRNAs with a high deviance in expression through bioinformatics pipeline 1 was used to select transcripts that were previously identified as preferentially expressed in either adrenal cortex or medulla from the proteinatlas.org [53]. Twenty-two transcripts were selected, 11 from adrenal medulla and 11 from adrenal cortex. The dataset was analyzed using Gene Set Enrichment Analysis version 9.09 on the gene pattern platform (https://genepattern.broadinstitute.org) [54,55]. Samples were normalized to log-scale and analyzed with default settings. 

### 4.7. Identification of Transcripts Shared between Cancer Types

The dataset comprising 2262 genes identified through extraction of mRNAs with a high deviance in expression through bioinformatics pipeline 1 was used. Genes with area under the Receiver Operating Characteristic (ROC) curve (Harrell’s C-statistic) of >0.9 for ACC, GBM, LGG, PPGL, NBL, and PNET versus remaining tumor types were selected. Difference between median gene expression value in ACC compared to (1) GBM, LGG, NBL, PNET and PPGL, and (2) remaining tumors were determined. Genes with fold change in ACC of <0.1 (compared to GBM, LGG, NBL, PNET and PPGL) and <2 (compared to remaining tumor types) were excluded. Remaining genes were annotated for overlapping gene ontology annotations version 2018-05-07 (https://www.ebi.ac.uk/QuickGO/).

## 5. Conclusions

ACC was a homogenous molecular group that showed a surprising association with neural crest derived tumors. PPGL mixed with both pancreatic NETs and NBL. Thus, the unbiased gene-expression analysis did not fully overlap with current clinicopathological classification of these tumors. In line with previous results, PPGL clustered together with other neural crest derived neoplasms.

## Figures and Tables

**Table 1:** Samples included from the Cancer Genome Atlas (TCGA) and Therapeutically Applicable Research To Generate Effective Treatments (TARGET). TCGA official nomenclature is shown in parentheses. *n*, number of cases included; ref, reference.

Cohort	Cohort, Full Name	*n*	Reference
ACC	Adrenocortical carcinoma	78	[5]
BLCA	Bladder urothelial carcinoma	100	[13,14]
BRCA	Breast invasive carcinoma	100	[15]
CESC	Cervical squamous cell carcinoma and Endocervical adenocarcinoma	100	[16]
CHOL	Cholangiocarcinoma	44	[17]
COAD	Colon adenocarcinoma	100	[18]
DLBC	Lymphoid neoplasm diffuse large B-cell lymphoma	47	
ESCA	Esophageal carcinoma	100	[12]
GBM	Glioblastoma multiforme	100	[19,20]
HNSC	Head and neck squamous cell carcinoma	100	[21]
KICH	Kidney chromophobe	88	[22]
KIRC	Kidney renal clear cell carcinoma	100	[23]
KIRP	Kidney renal papillary cell carcinoma	100	[24]
LAML	Acute myeloid leukemia	100	[25]
LGG	Brain Lower Grade Glioma	100	[19,26]
LIHC	Liver hepatocellular carcinoma	100	[27]
LUAD	Lung adenocarcinoma	100	[28]
LUSC	Lung squamous cell carcinoma	100	[29]
MESO	Mesothelioma	85	
OV	Ovarian serous cystadenocarcinoma	100	[30]
PAAD	Pancreatic adenocarcinoma	100	[31]
PNET (PAAD)	Pancreatic neuroendocrine tumor	8	[31]
PPGL (PCPG)	Pheochromocytoma and paraganglioma	179	[9]
PRAD	Prostate adenocarcinoma	100	[32]
READ	Rectum adenocarcinoma	100	[18]
SARC	Sarcoma	100	[33]
SKCM	Skin cutaneous melanoma	100	[34]
STAD	Stomach adenocarcinoma	100	[35]
TGCT	Testicular germ cell tumors	100	[36]
THCA	Thyroid carcinoma	100	[37]
THYM	Thymoma	100	[38]
UCEC	Uterine corpus endometrial Carcinoma	100	[39]
UCS	Uterine carcinosarcoma	55	[40]
UVM	Uveal melanoma	79	[41]
NBL	Neuroblastoma	156	[42]
Total		3319	

## Supplementary Materials

The following are available online at http://www.mdpi.com/2072-6694/10/12/518/s1, Figure S1: Bioinformatics pipeline 1. Unsupervised hierarchal clustering based on transcriptome data from 35 TCGA tumor categories with adrenal tumors annotated accordingly to molecular subtype, Figure S2: Bioinformatics pipeline 2. 1975 transcripts selected. Unsupervised clustering of 35 TCGA tumor categories with adrenal tumors annotated accordingly to molecular subtype, Figure S3: Bioinformatics pipeline 1. Unsupervised hierarchal clustering of 3319 samples annotated for TCGA tumor category with selected tumors annotated accordingly to molecular subtype, Figure S4: (A) Bioinformatics pipeline 2. 1975 transcripts selected. Unsupervised hierarchal clustering of 3319 samples annotated for TCGA tumor category with selected tumors annotated accordingly to molecular subtype. (B) Bioinformatics pipeline 2. Principal component analysis of 3319 samples with selected tumors annotated for TCGA or TARGET tumor category, Figure S5: Bioinformatics pipeline 1. Unsupervised hierarchal clustering of all ACC and SARC samples available in the TCGA database, Figure S6: Bioinformatics pipeline 1. Unsupervised clustering of 35 TCGA tumor categories as well as 8 PAAD samples annotated as PNET, Figure S7: Bioinformatics pipeline 2. 1975 transcripts selected. Unsupervised clustering of 35 TCGA tumor categories as well as 8 PAAD samples annotated as PNET, Figure S8: Bioinformatics pipeline 1. Unsupervised hierarchal clustering based on transcriptome data from 35 TCGA tumor categories with adrenal tumors annotated accordingly to molecular subtype. All samples in the PPGL cohort labeled as pheochromocytoma were removed, Figure S9: Consensus matrices of unsupervised cluster of cluster classification of 3319 TCGA and TARGET samples, Figure S10: Bioinformatics pipeline 1. Unsupervised hierarchal clustering of all PPGL as well as 8 PAAD samples annotated as PNET, Figure S11: Unsupervised hierarchal clustering of GBM, LGG, NBL, PNET and PPGL (minus cortical admixture subgroup), Table S1a: Genes preferentially expressed in Adrenocortical carcinoma (ACC) as well in as neural crest tumors (glioblastoma, low grade glioma, neuroblastoma, pancreatic neuroendocrine tumor and pheochromocytoma and paraganglioma), Table S1b: Go-enrichment analysis of genes co-expressed in Adrenocortical carcinoma (ACC) and glioma (glioblastoma and low grade glioma) and neuroendocrine tumors (neuroblastoma, pancreatic neuroendocrine tumor and pheochromocytoma and paraganglioma).

## Abbreviations

Abbrev.	Defination
TCGA	the Cancer Genome Atlas
TARGET	Therapeutically Applicable Research to Generate Effective Treatments
ACC	Adrenocortical carcinoma
PPGL	pheochromocytoma and paraganglioma
PNET	pancreatic neuroendocrine tumor
NBL	Neuroblastoma
COC	Cluster of Clusters
BRCA	Breast invasive carcinoma
BLCA	Bladder urothelial carcinoma
CESC	Cervical squamous cell carcinoma and Endocervical adenocarcinoma
CHOL	Cholangiocarcinoma
COAD	Colon adenocarcinoma
DLBC	Lymphoid neoplasm diffuse large B-cell lymphoma
ESCA	Esophageal carcinoma
GBM	Glioblastoma multiforme
HNSC	Head and neck squamous cell carcinoma
KICH	Kidney chromophobe
KIRC	Kidney renal clear cell carcinoma
KIRP	Kidney renal papillary cell carcinoma
LAML	Acute myeloid leukemia
LGG	Brain Lower Grade Glioma
LIHC	Liver hepatocellular carcinoma
LUAD	Lung adenocarcinoma
LUSC	Lung squamous cell carcinoma
MESO	Mesothelioma
OV	Ovarian serous cystadenocarcinoma
PAAD	Pancreatic adenocarcinoma
PRAD	Prostate adenocarcinoma
READ	Rectum adenocarcinoma
SARC	Sarcoma
SKCM	Skin cutaneous melanoma
STAD	Stomach adenocarcinoma
TGCT	Testicular germ cell tumors
THCA	Thyroid carcinoma
THYM	Thymoma
UCEC	Uterine corpus endometrial Carcinoma
UCS	Uterine carcinosarcoma
UVM	Uveal melanoma
