# Supplementary material for: RNA-Sequencing Analysis of Adrenocortical Carcinoma, Pheochromocytoma and Paraganglioma from a Pan-Cancer Perspective
Source: Cancers (Basel). 2018 Dec 15;10(12):518. doi: 10.3390/cancers10120518 (PMC6315481; doi:10.3390/cancers10120518)
Supplement: Supplementary file 1 [file cancers-10-00518-s001.pdf]

cancers

Supplementary Materials: RNA-Sequencing Analysis of Adrenocortical Carcinoma, Pheochromocytoma and Paraganglioma from a Pan-Cancer Perspective

Joakim Crona, Samuel Backman, Staffan Welin, David Taïeb, Per Hellman, Peter Stålberg, Britt Skogseid and Karel Pacak

Abbreviations:

Abbrev.	Defination
**TCGA Nomenclature**	
TCGA	the Cancer Genome Atlas
ACC	Adrenocortical carcinoma
BLCA	Bladder urothelial carcinoma
BRCA	Breast invasive carcinoma
CESC	Cervical squamous cell carcinoma and endocervical adenocarcinoma
CHOL	Cholangiocarcinoma
COAD	Colon adenocarcinoma
DLBC	Lymphoid neoplasm diffuse large B-cell lymphoma
ESCA	Esophageal carcinoma
GBM	Glioblastoma multiforme
HNSC	Head and neck squamous cell carcinoma
KICH	Kidney chromophobe
KIRC	Kidney renal clear cell carcinoma
KIRP	Kidney renal papillary cell carcinoma
LAML	Acute myeloid leukemia
LGG	Brain lower grade glioma
LIHC	Liver hepatocellular carcinoma
LUAD	Lung adenocarcinoma
LUSC	Lung squamous cell carcinoma
MESO	Mesothelioma
OV	Ovarian serous cystadenocarcinoma
PAAD	Pancreatic adenocarcinoma
PRAD	Prostate adenocarcinoma
READ	Rectum adenocarcinoma
SARC	Sarcoma
SKCM	Skin cutaneous melanoma
STAD	Stomach adenocarcinoma
TGCT	Testicular germ cell tumors
THCA	Thyroid carcinoma
THYM	Thymoma
UCEC	Uterine corpus endometrial carcinoma
UCS	Uterine carcinosarcoma
UVM	Uveal melanoma
**Non TCGA Nomenclature**	
CoC	Cluster of clusters
NBL	Neuroblastoma
NET	Neuroendocrine tumor
PPGL	Pheochromocytoma and Paraganglioma
Cortical	Cortical admixture PPGL
Hypoxia	Pseudohypoxic PPGL
Kinase	Kinase signaling PPGL
PCC	Pheochromocytoma
PGL	Paraganglioma
TARGET	Therapeutically Applicable Research to Generate Effective Treatments
Wnt	wnt-altered PPGL

Supplementary figure 1A, 1417 genes, Molecular subtypes

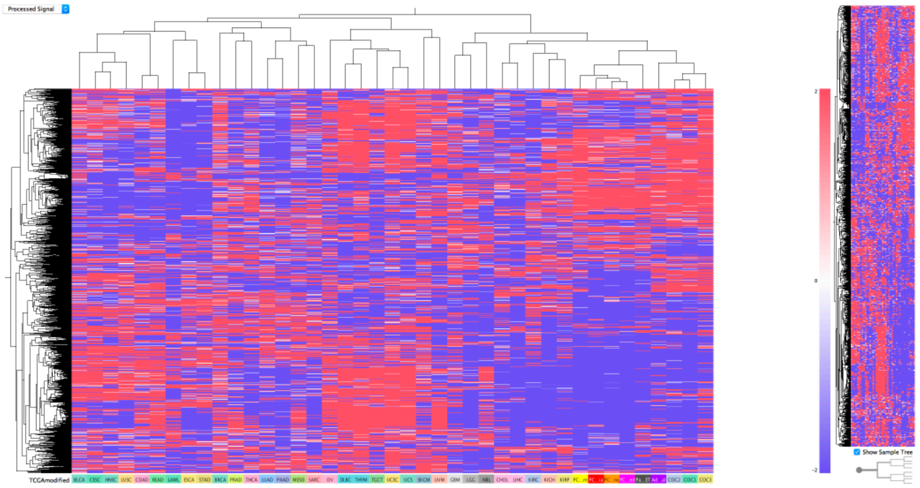

Supplementary figure 1B, 2248 genes, Molecular subtypes

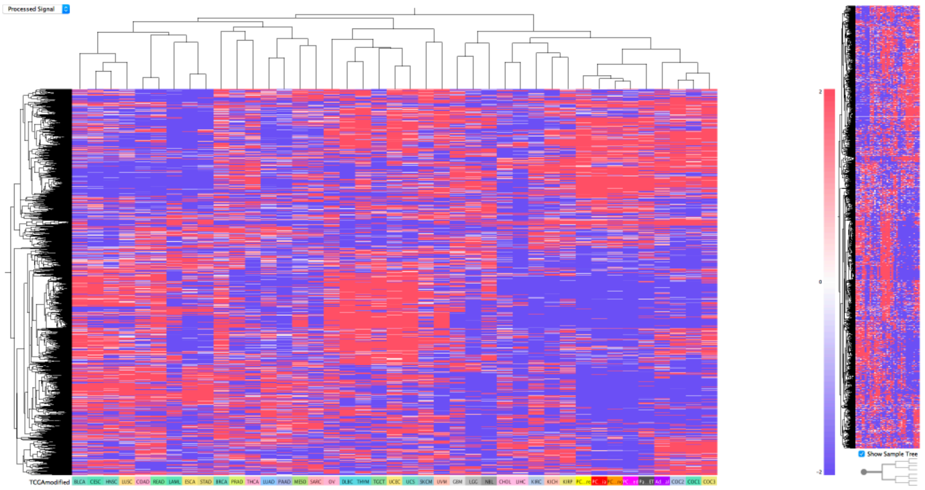

Supplementary figure 1C, 4598 genes, Molecular subtypes

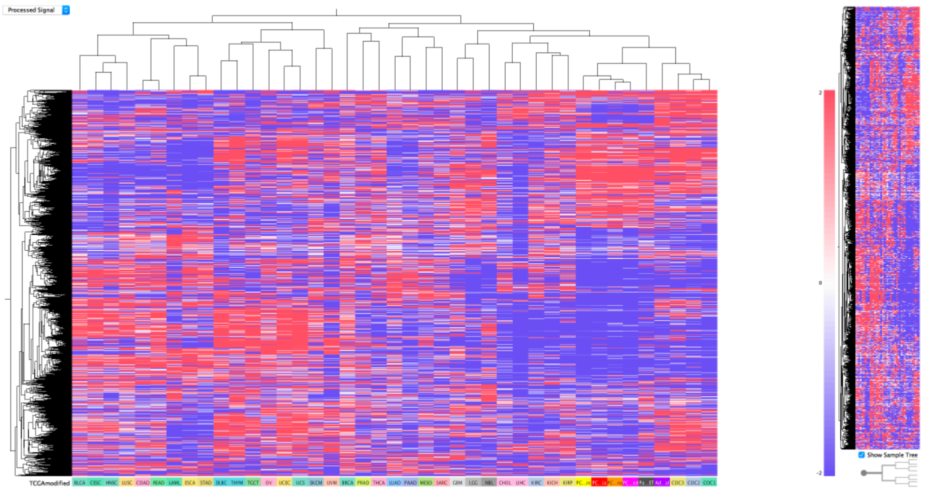

Supplementary Figure S1. Bioinformatics pipeline 1. Unsupervised hierarchal clustering based on transcriptome data from 35 TCGA tumor categories with adrenal tumors annotated accordingly to molecular subtype.

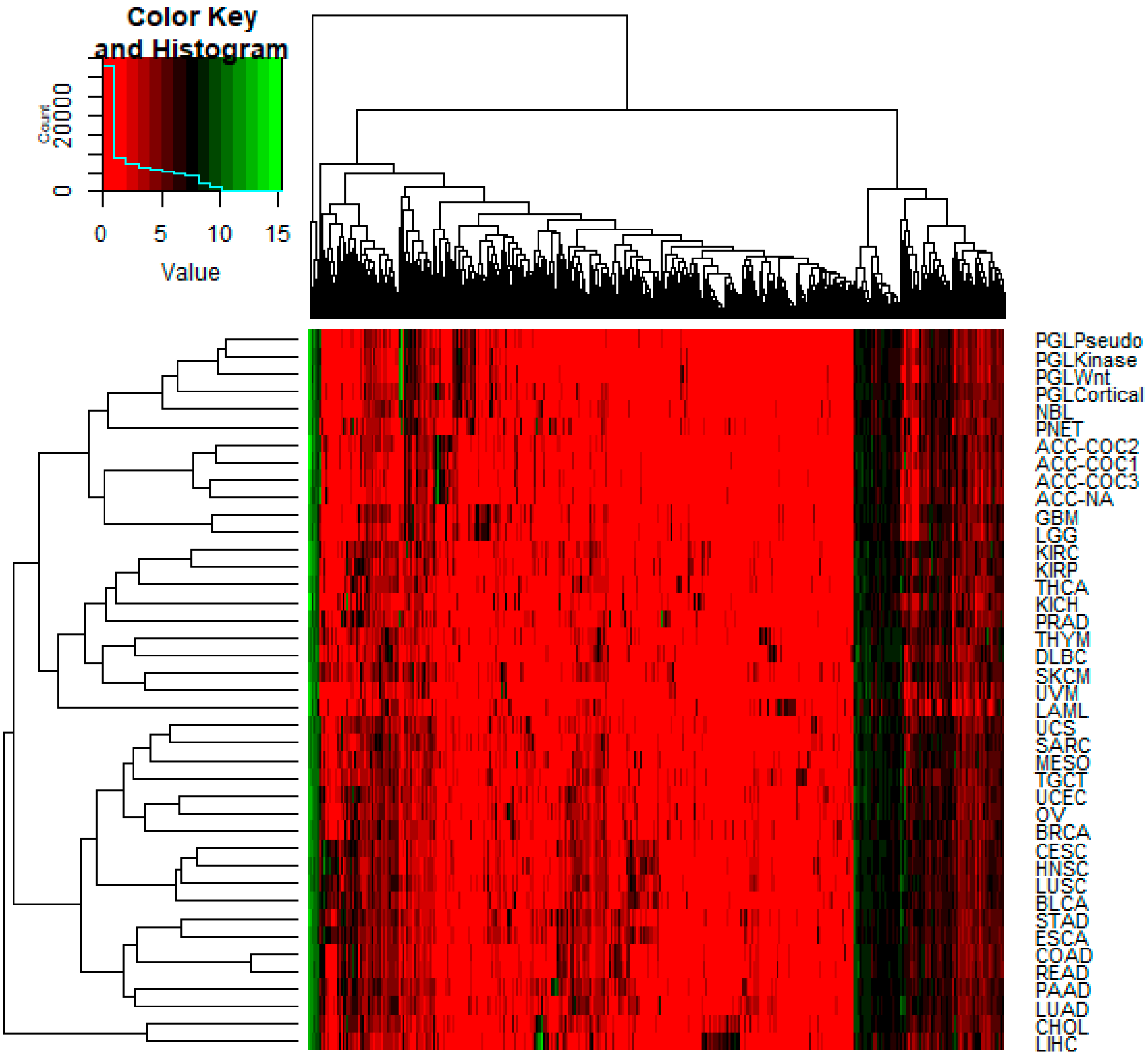

Supplementary Figure S2. Bioinformatics pipeline 2. 1975 transcripts selected. Unsupervised clustering of 35 TCGA tumor categories with adrenal tumors annotated accordingly to molecular subtype.

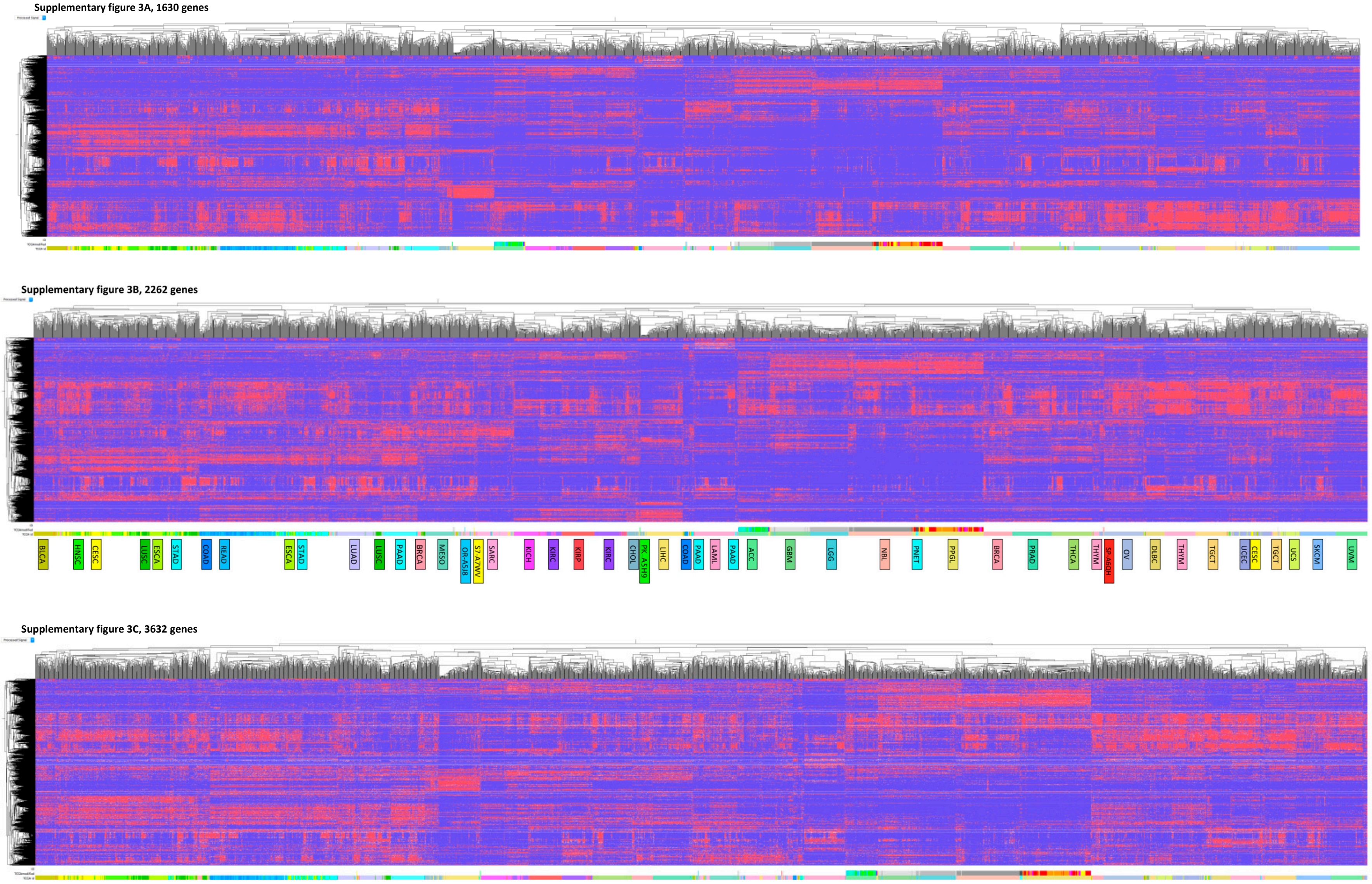

Supplementary Figure S3. Bioinformatics pipeline 1. Unsupervised hierarchal clustering of 3319 samples annotated for TCGA tumor category with selected tumors annotated accordingly to molecular subtype.

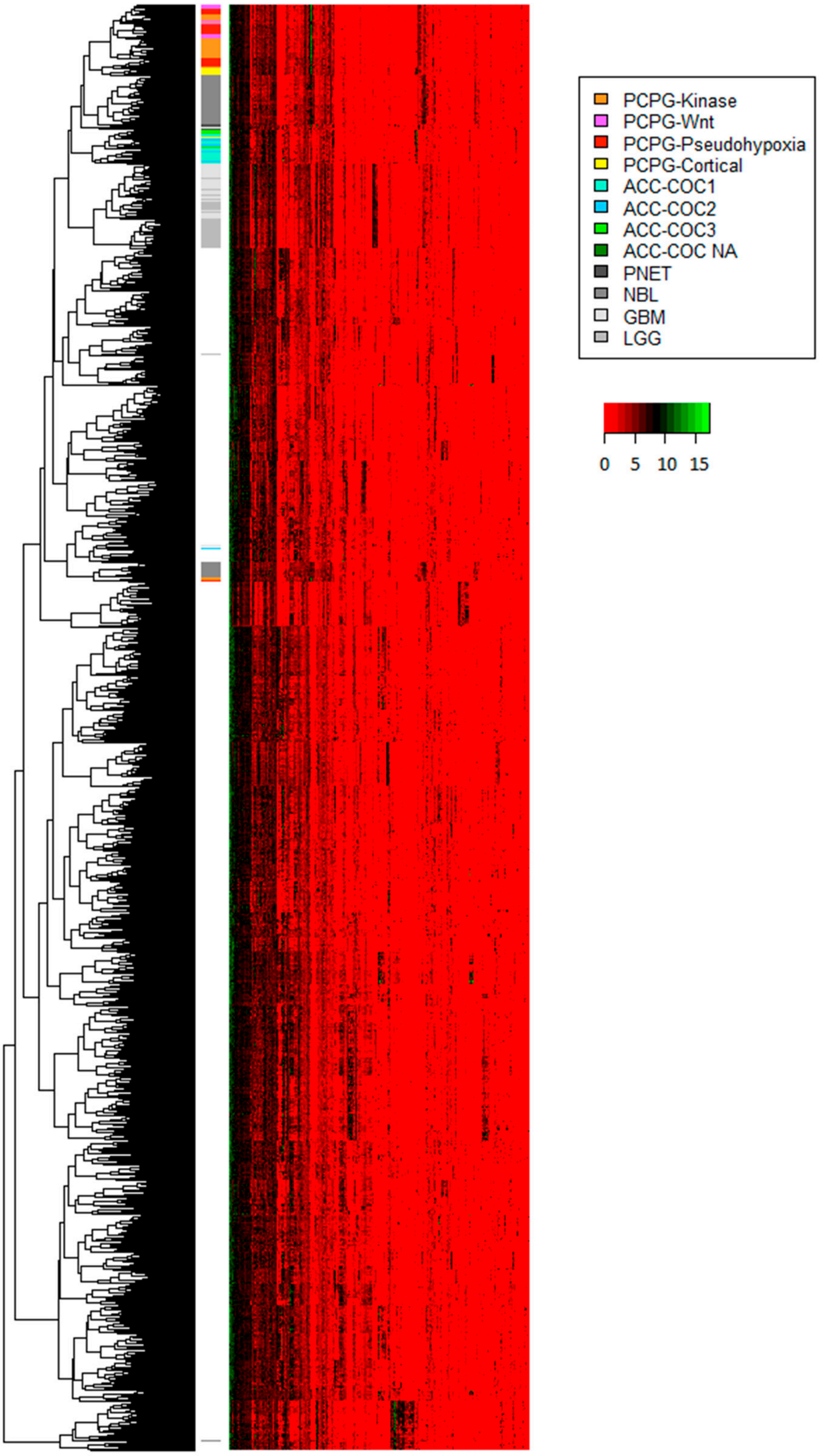
PCPG-Kinase
PCPG-Wnt
PCPG-Pseudohypoxia
PCPG-Cortical
ACC-COC1
ACC-COC2
ACC-COC3
ACC-COC NA
PNET
NBL
GBM
LGG
0 5 10 15

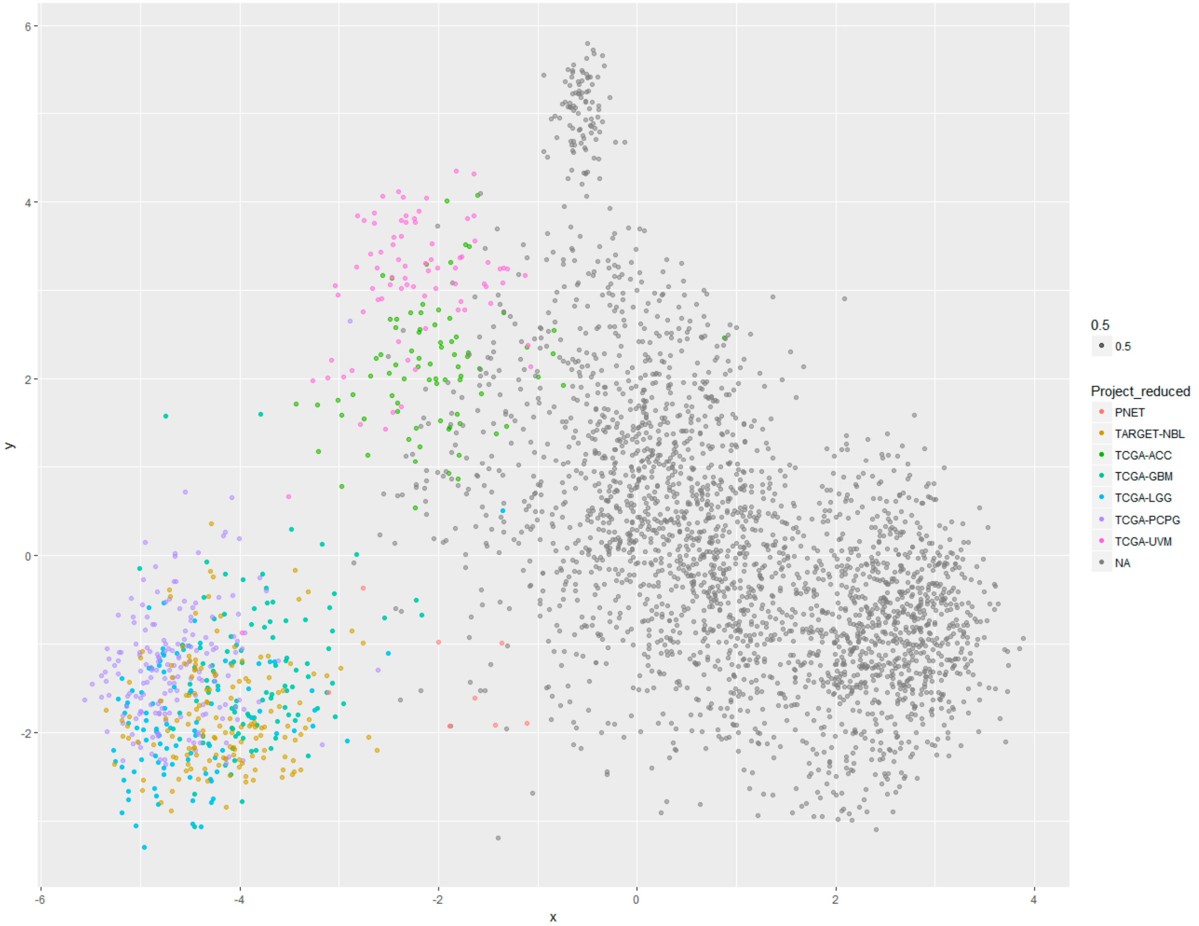

Supplementary Figure S4. (**A**) Bioinformatics pipeline 2. 1975 transcripts selected. Unsupervised hierarchal clustering of 3319 samples annotated for TCGA tumor category with selected tumors annotated accordingly to molecular subtype. (**B**) Bioinformatics pipeline 2. Principal component analysis of 3319 samples with selected tumors annotated for TCGA or TARGET tumor category.

Supplementary figure 5A, 1620 transcripts, SARC+ACC

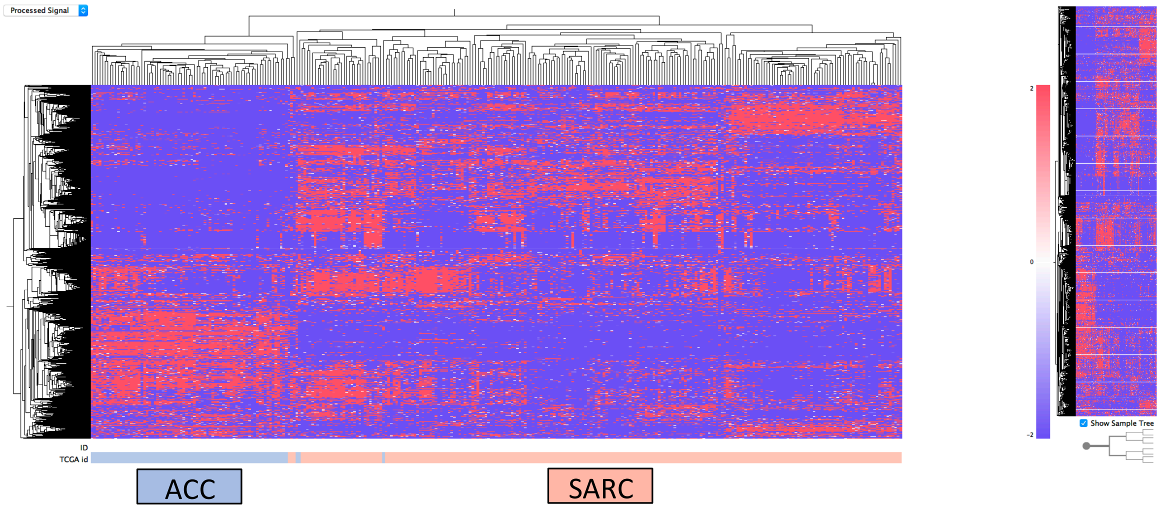

Supplementary figure 5B, 2638 transcripts, SARC+ACC

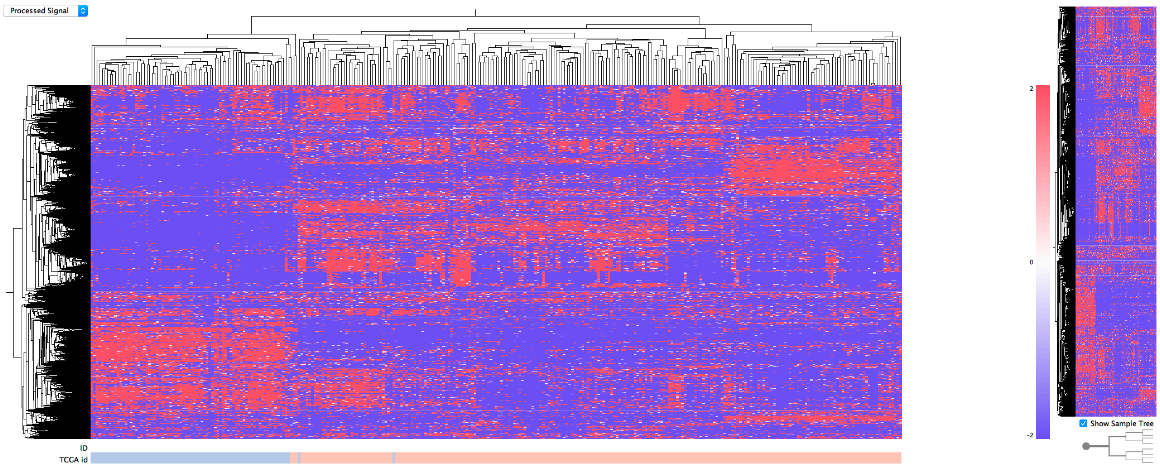

Supplementary figure 5C, 3918 transcripts, SARC+ACC

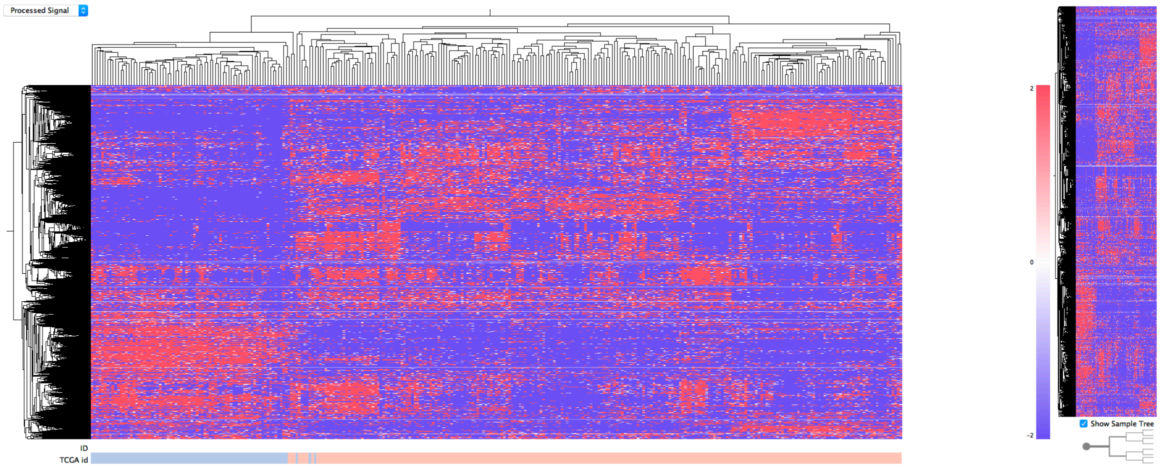

Supplementary Figure S5. Bioinformatics pipeline 1. Unsupervised hierarchal clustering of all ACC and SARC samples available in the TCGA database.

Supplementary figure 6A, 1390 genes, No molecular subtypes

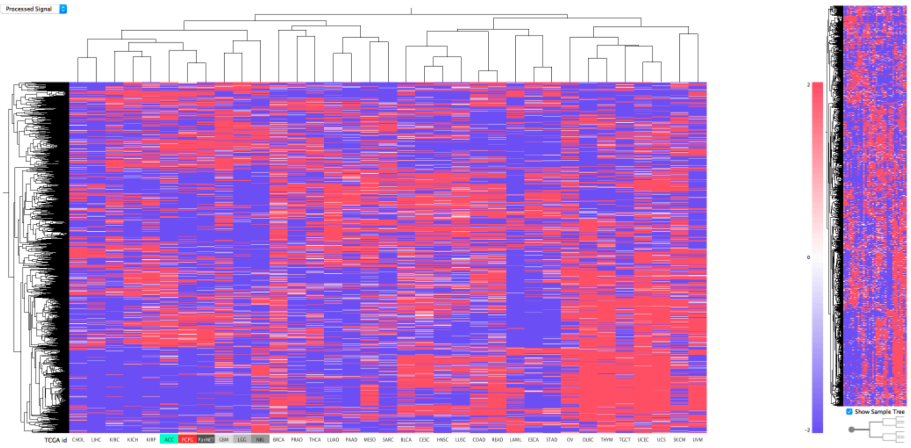

Supplementary figure 6B, 1946 genes, No molecular subtypes

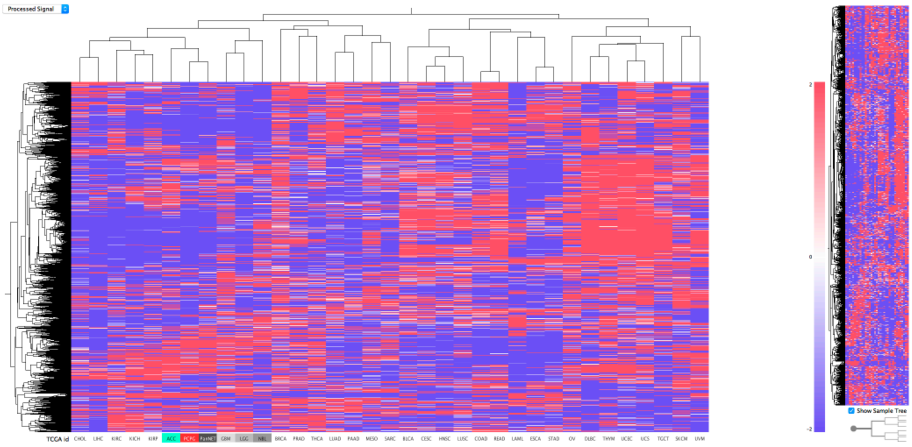

Supplementary figure 6C, 3111 genes, No molecular subtypes

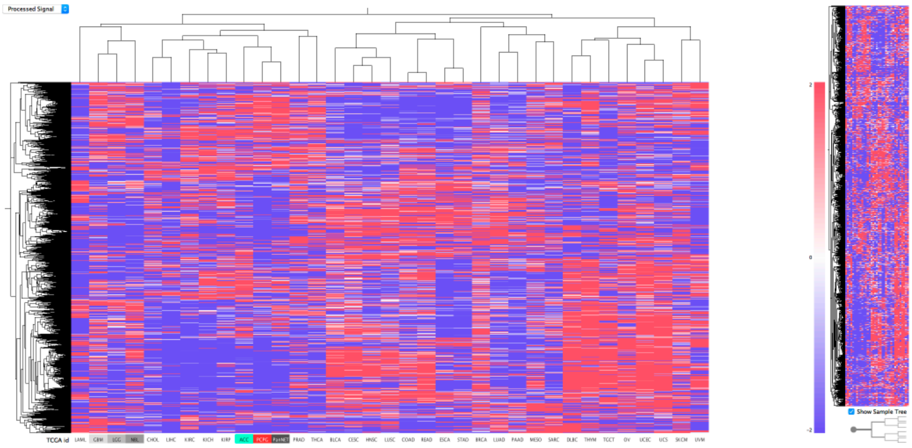

Supplementary Figure S6. Bioinformatics pipeline 1. Unsupervised clustering of 35 TCGA tumor categories as well as 8 PAAD samples annotated as PNET.

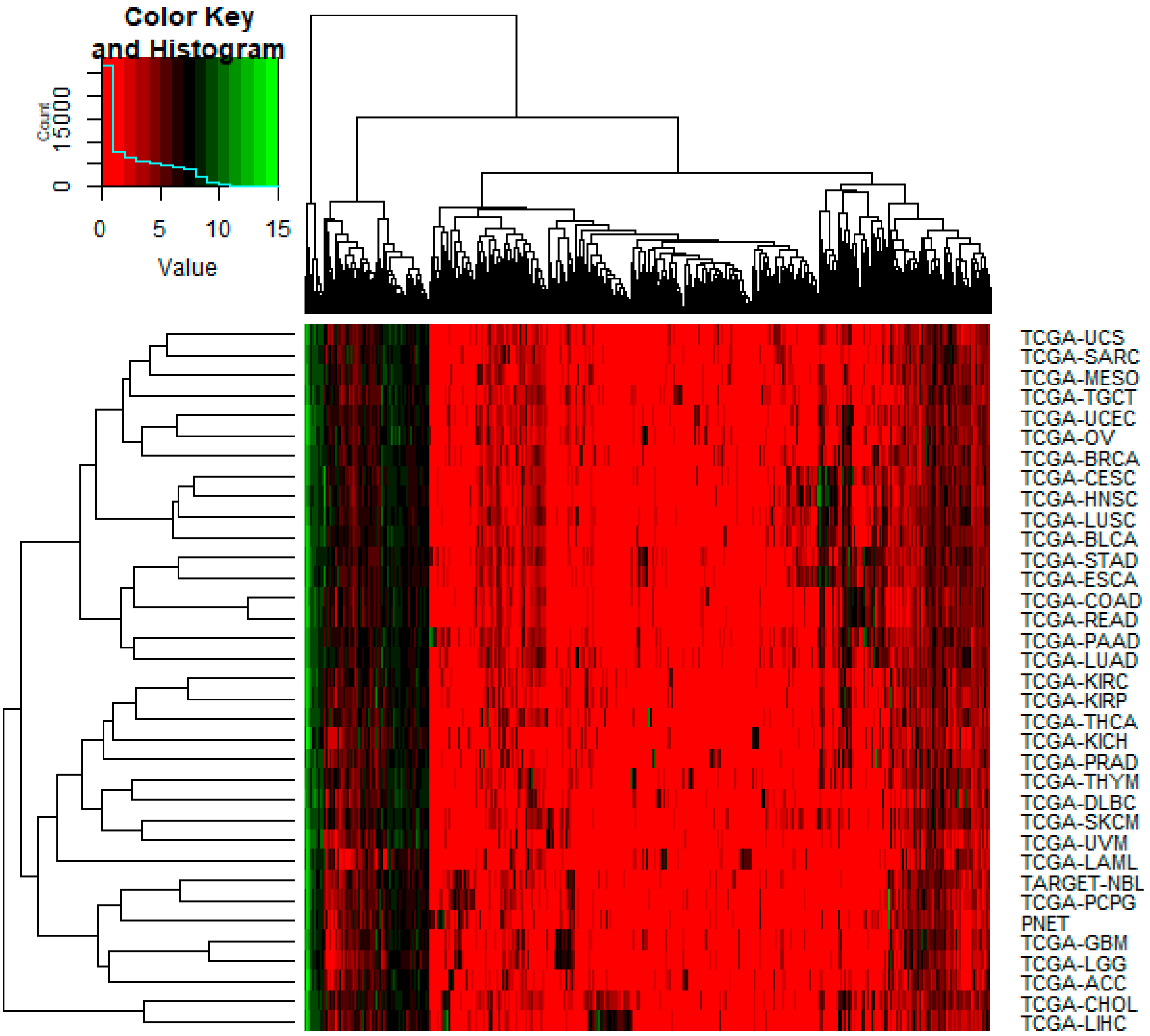

Supplementary Figure S7. Bioinformatics pipeline 2. 1975 transcripts selected. Unsupervised clustering of 35 TCGA tumor categories as well as 8 PAAD samples annotated as PNET.

Supplementary Figure 8, 1941 transcripts, Molecular subtypes Pheochromocytoma samples removed

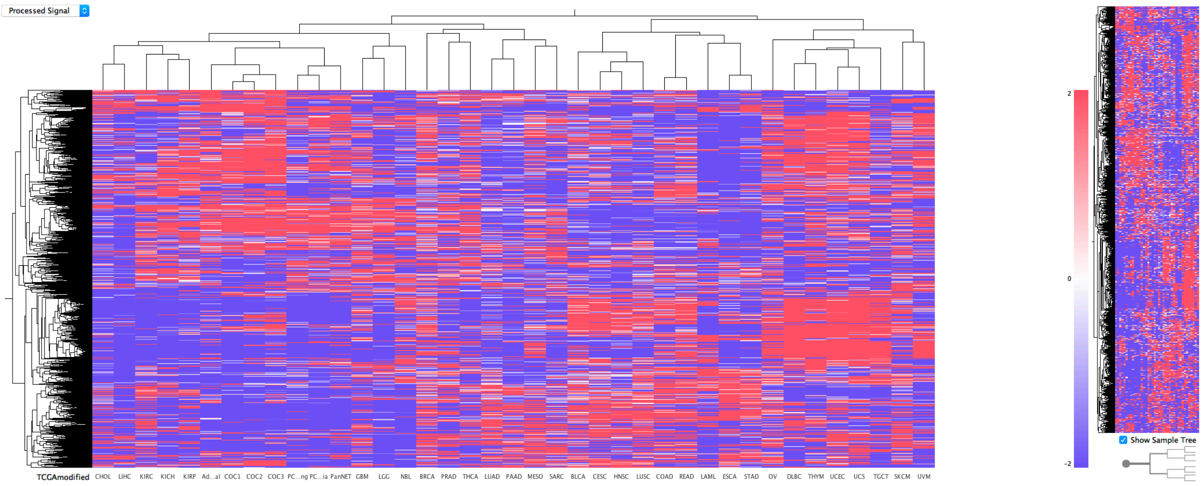

Supplementary Figure S8. Bioinformatics pipeline 1. Unsupervised hierarchal clustering based on transcriptome data from 35 TCGA tumor categories with adrenal tumors annotated accordingly to molecular subtype. All samples in the PPGL cohort labeled as pheochromocytoma were removed.

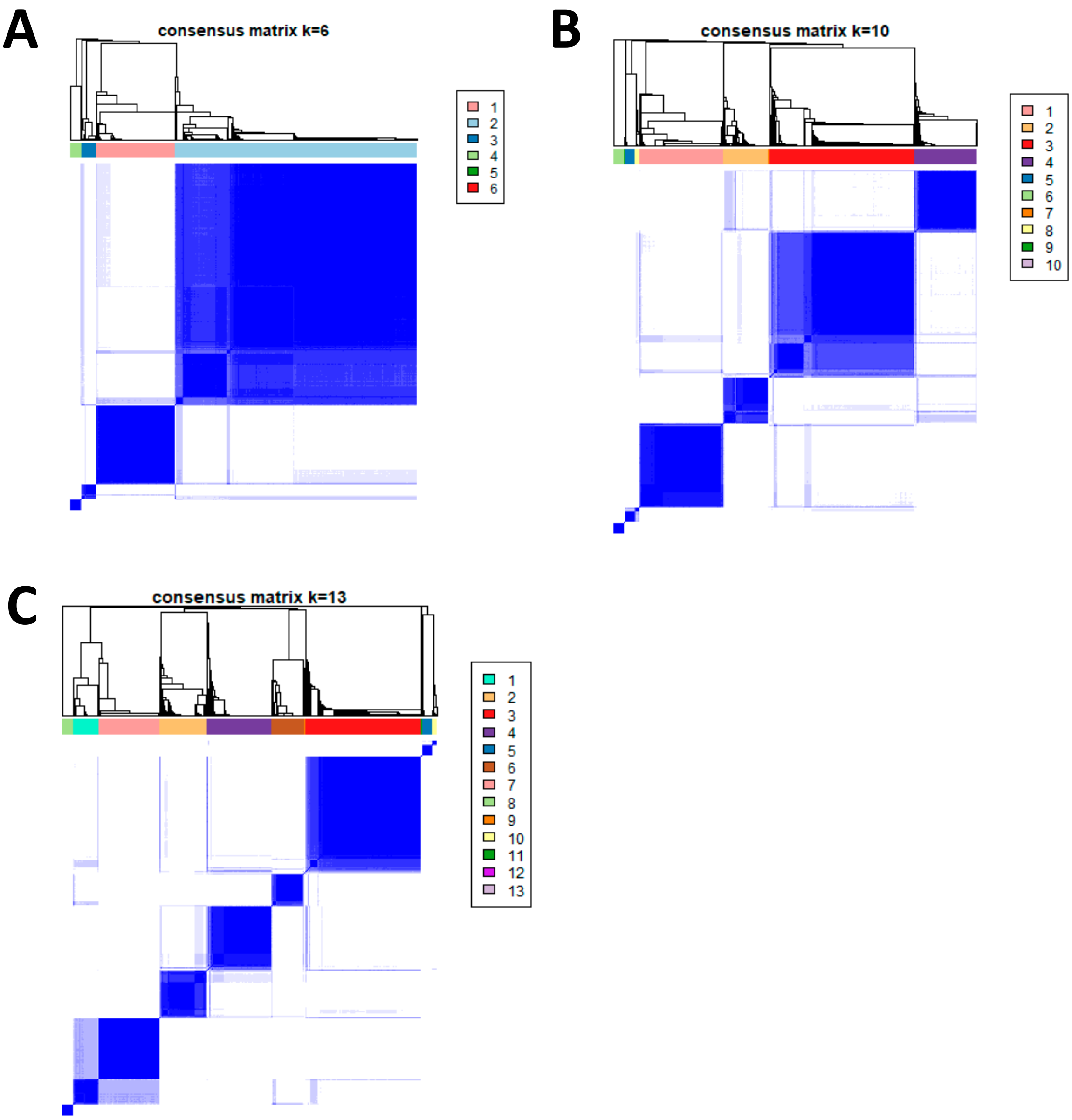

Supplementary Figure S9. Consensus matrices of unsupervised cluster of cluster classification of 3319 TCGA and TARGET samples.

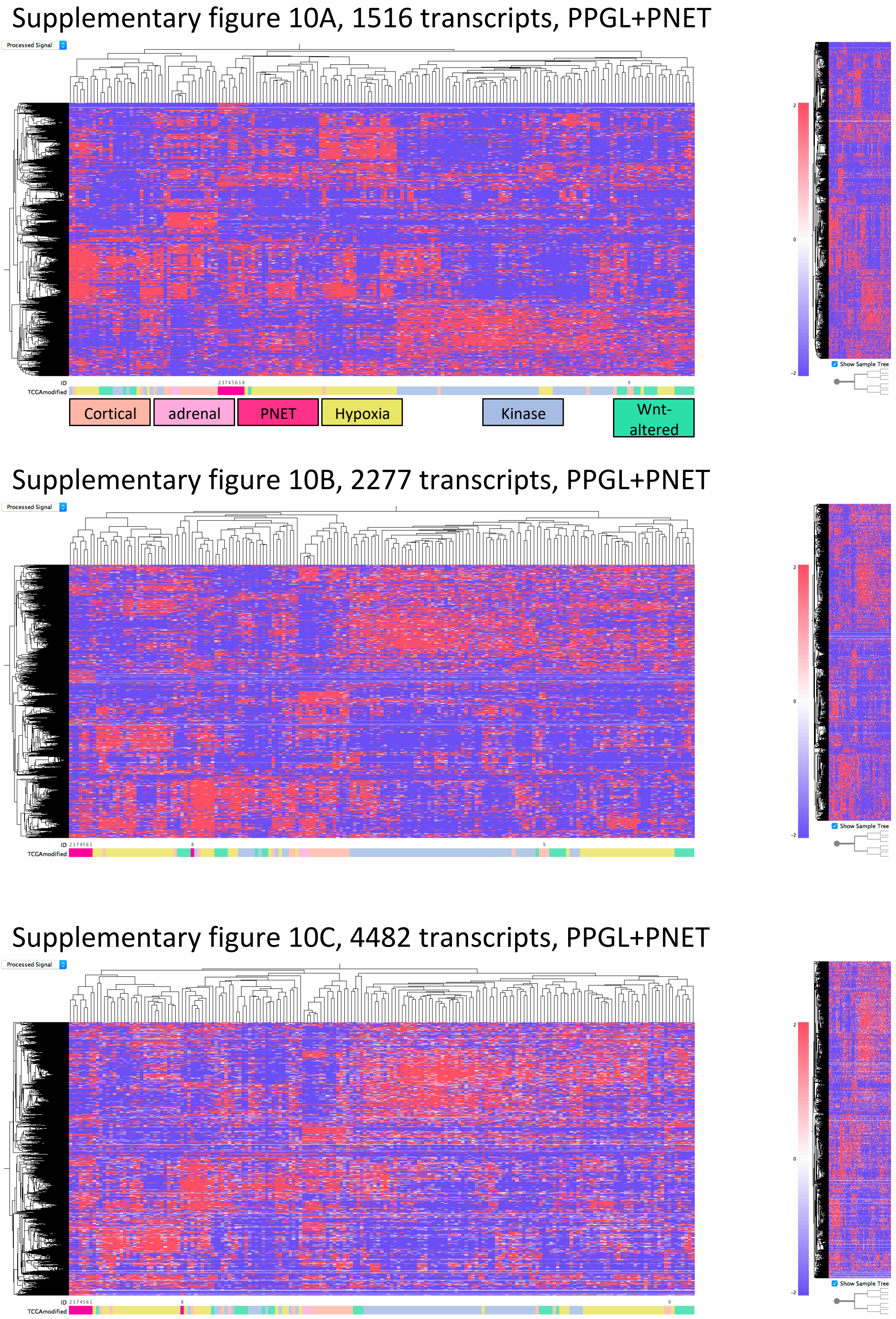

Suppelementary Figure S10. Bioinformatics pipeline 1. Unsupervised hierarchal clustering of all PPGL as well as 8 PAAD samples annotated as PNET.

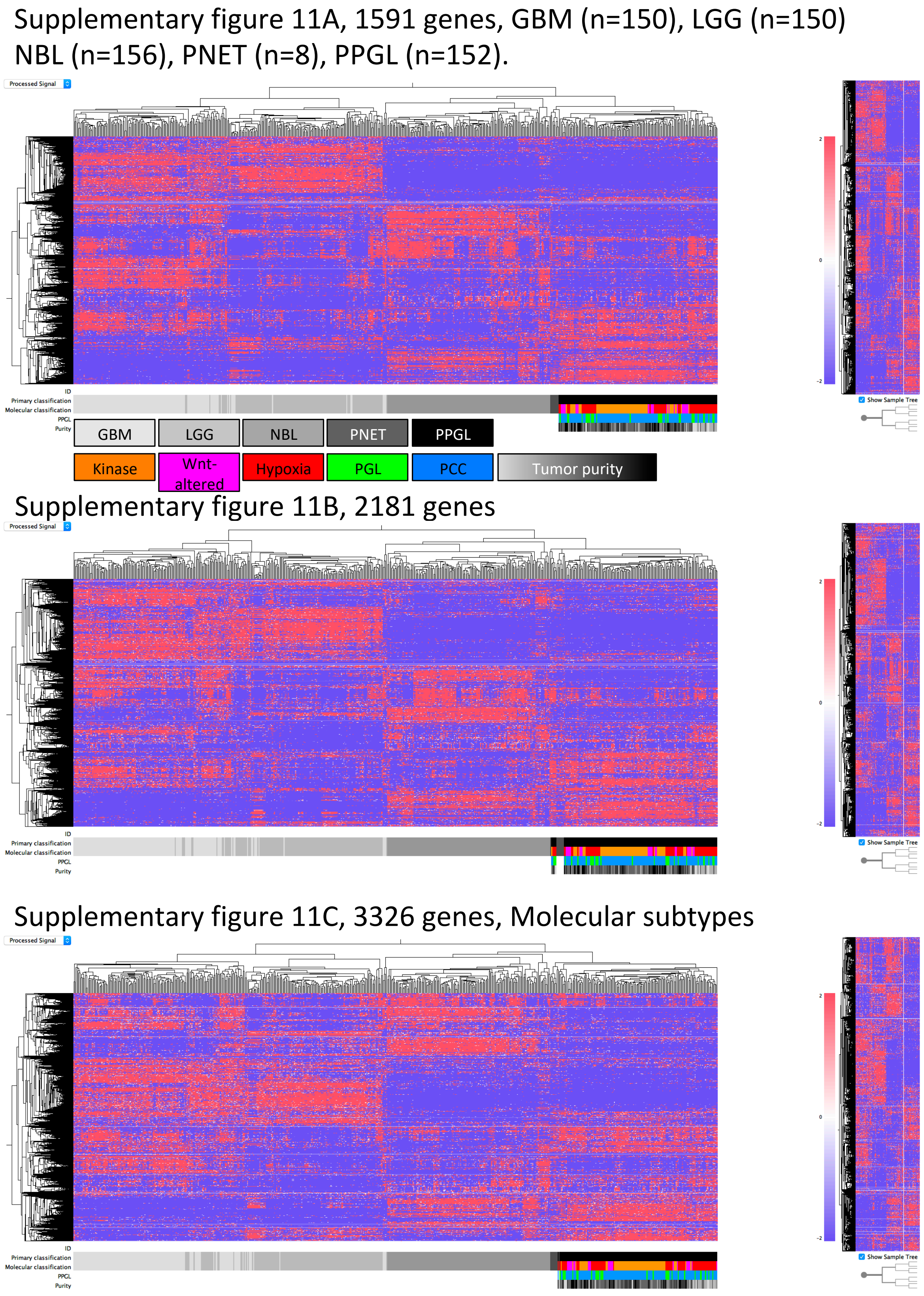

Supplementary Figure S11. Unsupervised hierarchal clustering of GBM, LGG, NBL, PNET and PPGL (minus cortical admixture subgroup).

Table S1a. Genes preferentially expressed in Adrenocortical carcinoma (ACC) as well in as neural crest tumors (glioblastoma, low grade glioma, neuroblastoma, pancreatic neuroendocrine tumor and pheochromocytoma and paraganglioma).

Gene Name	ACC, Mean	Glioblastoma, Mean	Low Grade Glioma, Mean	Pheochromocytoma and Paraganglioma, Mean	Neuroblastoma, Mean	Pancreatic Neuroendocrine Tumor, Mean	Remaining Tumors, Mean	ACC fold Difference to Non Neural Crest Tumors	ACC fold Difference to Neural Crest Tumors	Area Under the Curve
SYP	39.5	13.4	35.7	132.2	51.5	82.0	0.9	59.74	0.56	0.989
DNER	31.0	74.5	137.1	66.2	17.7	16.3	1.4	116.40	0.34	0.977
BEX1	105.7	104.8	205.5	396.6	254.0	386.0	7.9	112.33	0.28	0.975
NDRG4	43.3	22.5	48.8	86.2	47.0	13.0	2.1	25.28	0.36	0.975
TERF2IP	49.3	38.9	64.7	116.7	75.7	74.2	21.9	2.37	0.67	0.967
TSPAN7	75.0	67.3	152.5	74.5	53.0	58.3	9.1	18.02	0.92	0.965
CHGB	113.3	5.0	32.6	7914.0	353.4	2446.9	5.2	266.45	0.24	0.958
VWA5B2	40.3	0.6	1.7	86.1	9.3	45.7	0.3	190.60	3.22	0.957
VAT1L	18.1	8.2	25.5	89.6	60.7	19.9	1.5	19.24	0.11	0.952
EEF1A2	153.3	59.9	67.7	550.2	447.2	347.9	20.9	46.06	0.24	0.937
STMN3	52.6	74.6	104.7	112.3	131.8	103.7	21.3	2.85	0.33	0.934
QDPR	22.5	35.0	44.1	150.2	40.2	94.0	14.2	2.11	0.48	0.924
ENO2	31.1	60.4	75.0	119.4	128.1	103.9	16.2	2.25	0.22	0.924
SARAF	192.2	163.3	167.7	242.9	163.6	262.4	82.6	2.37	0.98	0.916
RTL8C	169.2	192.0	178.7	202.5	126.8	111.2	78.2	2.25	0.92	0.904

Table S1b. Go-enrichment analysis of genes co-expressed in Adrenocortical carcinoma (ACC) and glioma (glioblastoma and low grade glioma) and neuroendocrine tumors (neuroblastoma, pancreatic neuroendocrine tumor and pheochromocytoma and paraganglioma).

GO-Term	GO-Name	Qualifier	Count	Percentage	Gene Names
GO:0005515	protein binding	enables	8	53	*EEF1A2, SARAF, ENO2, EEF1A2, VAT1L, CHGB, NDRG4, BEX1, DNER*
GO:0005737	cytoplasm	part_of	6	40	*ENO2, QDPR, STMN3, EEF1A2, NDRG4, BEX1*
GO:0016020	membrane	part_of	5	33	*SARAF, ENO2, TSPAN7, DNER, SYP*
GO:0016021	integral component of membrane	part_of	4	27	*SARAF, TSPAN7, DNER, SYP*
GO:0043025	neuronal cell body	part_of	3	20	*ENO2, EEF1A2, DNER*
GO:0043005	neuron projection	part_of	3	20	*QDPR, STMN3, SYP*
GO:0005829	cytosol	part_of	3	20	*ENO2, QDPR, NDRG4*
GO:0016491	oxidoreductase activity	enables	2	13	*QDPR, VAT1L*
GO:0055114	oxidation-reduction process	involved_in	2	13	*QDPR, VAT1L*
GO:0030154	cell differentiation	involved_in	2	13	*NDRG4, BEX1*
GO:0006897	endocytosis	involved_in	2	13	*DNER, SYP*
GO:0070062	extracellular exosome	part_of	2	13	*ENO2, QDPR*
GO:0043209	myelin sheath	part_of	2	13	*EEF1A2, ENO2*
GO:0005886	plasma membrane	part_of	2	13	*ENO2, DNER,*
GO:0005789	endoplasmic reticulum membrane	part_of	2	13	*SARAF, NDRG4*
